# A Rare Case of Viridans Group Streptococci Pyogenic Liver Abscess in a Young Male With No Prior Risk Factors

**DOI:** 10.7759/cureus.37721

**Published:** 2023-04-17

**Authors:** Menachem Jacobs, Moshe Weber, Marc Ganz, Avrohom Karp, Treven Wesley, Daniel Miller

**Affiliations:** 1 Public Health Sciences, State University of New York Downstate Health Sciences University, Brooklyn, USA; 2 Internal Medicine, State University of New York Downstate Health Sciences University, Brooklyn, USA; 3 Health Sciences, New York Medical College, Valhalla, USA; 4 Internal Medicine, Icahn School of Medicine at Mount Sinai, Queens Hospital Center, Queens, USA

**Keywords:** general internal medicine, infectious disease, hepatology, viridans streptococci, pyogenic liver abscess (pla)

## Abstract

Pyogenic liver abscesses (PLAs) secondary to bacterial etiologies are rare in North America and other developed countries. The predominant etiology of PLAs is an infection extending from the hepatobiliary or intestinal system. As such, the most common pathogens isolated from PLA in the United States are *Escherichia coli* and *Klebsiella*. Viridans group streptococci (VGS), on the other hand, are a large group of commensal bacteria in the oral flora and are a significantly less common cause of infection. Here, we report a rare case of a complicated isolated VGS PLA in a patient without known comorbidities. The patient was born and raised in the United States without recent travel history. Computed tomography (CT) with contrast showed multiple hypodense multiloculated lesions in the right lobe of the liver, measuring up to 13 cm, with mild wall thickening of the distal ileum and cecum. The abscesses were confirmed later as *Streptococcus viridans* PLA. The patient was treated with CT-guided drainage and IV antibiotics and, after that, made a quick recovery and was discharged. Our case underlines the significance of considering liver abscess as a differential even in previously healthy individuals with no known prior comorbid conditions, as prompt recognition is imperative in preventing morbidity and mortality.

## Introduction

Pyogenic liver abscesses (PLAs) are uncommon in healthy individuals and are often associated with underlying hepatobiliary or intestinal infections [1]. PLAs are most often seen in developing countries and are far less frequent in North America, with an incidence of approximately 2.3 cases per 100,000, are associated with significant morbidity, and have a mortality risk between 6-10% [2]. The predominant etiology of PLAs in the United States is *Escherichia coli *and *Klebsiella*, while Viridans group streptococci (VGS) are most commonly a part of human oral flora living in close association with the gingiva, and a rare cause [3,4].

## Case presentation

A 28-year-old male with no significant past medical history presented to the emergency department with complaints of dull, intermittent right upper quadrant pain that he rated 7/10 for two weeks, associated with non-bloody, non-bilious emesis (one to two episodes a day), subjective fever, shortness of breath, and decreased appetite. He reported unquantifiable weight loss but denied night sweats, sick contacts, and recent travel history. Social history was insignificant. 

On presentation, the patient was febrile with a temperature of 103°F, tachycardic with a heart rate of 146 beats per minute, respiratory rate of 22, and hypertensive with a blood pressure of 143/86 mmHg. He was noted to be alert, awake, oriented, and in mild distress. Ocular examination showed it was anicteric. Throat and neck examinations were benign, with no oral lesions or lymphadenopathy. Chest examination revealed regular heart sounds, and lung sounds were also normal and vesicular. His abdomen was soft and distended, with tenderness noted in all quadrants. Murphy’s sign was negative. Bowel sounds were normal in all quadrants. No masses were palpated otherwise.

Laboratory workup revealed anemia with hemoglobin (Hgb) of 7.7 g/dL, leukocytosis with a white blood cell count of 16,000/μL, platelets 483 K/uL, normal blood urea nitrogen (BUN), and elevated creatinine of 1.4 mg/dL. Liver enzymes were grossly normal with a normal alanine aminotransferase (ALT) level of 13 U/L, an elevated aspartate aminotransferase (AST) of 52 U/L, and alkaline phosphatase that was within a normal range. Total bilirubin was 1.7 mg/dL, and lactate was elevated at 3.4 mmol/L. The patient was also tested for hepatitis B and C markers, which were negative. The patient's initial laboratory findings are summarized in (Table 1).

**Table 1:** Initial Laboratory Data L: low; H: high; BUN: blood urea nitrogen; AST: aspartate aminotransferase; ALT: alanine transaminase; eGFRcr: estimated glomerular filtration rate-creatinine, MPV: mean platelet volume; Hgb: hemoglobin; HCT: hematocrit; MCV: mean corpuscular volume; INR: international normalized ratio; aPTT: activated partial thromboplastin time

Laboratory Test	Result	Reference Range
Sodium Level	132 (L)	136-145 mmol/L
Potassium Level	3.8	3.5-5.1 mmol/L
Chloride Level	91 (L)	98-107 mmol/L
CO2	31	21-31 mmol/L
Glucose Random	107	70-99 mg/dL
BUN	13	7-25 mg/dL
Creatinine	1.4 (H)	0.7-1.3 mg/dL
Protein Total	7.1	6.0-8.3 g/dL
Albumin	2.8 (L)	3.5-5.7 g/dL
Alkaline Phosphatase	80	34-104 U/L
AST	52 (H)	13-39 U/L
ALT	13	7-52 U/L
Calcium Level	7.9 (L)	8.2- 10.0 mg/dL
Bilirubin Total	1.7 (H)	0.3-1.0 mg/dL
Bilirubin Direct	0.97 (H)	0.03-0.18 mg/dL
Anion Gap	14	10-20 mmol/L
eGFRcr	70	>=60 ml/min/1.73 m2
WBC	16.76 (H)	3.50-10.80 K/uL
MPV	10.2	7.5-11.5 fL
RBC	2.87	4.70-6.10 M/uL
Hgb	7.7 (L)	14.0-18.0 g/dL
HCT	24.4 (L)	42.0-52.0%
MCV	85.0	[80.0 - 95.0 fL]
Prothrombin time	20.5 (H)	[10.8-13.7sec]
INR	1.6	<2
aPTT	31.6	23.5-35.5 sec

**Figure 1:**
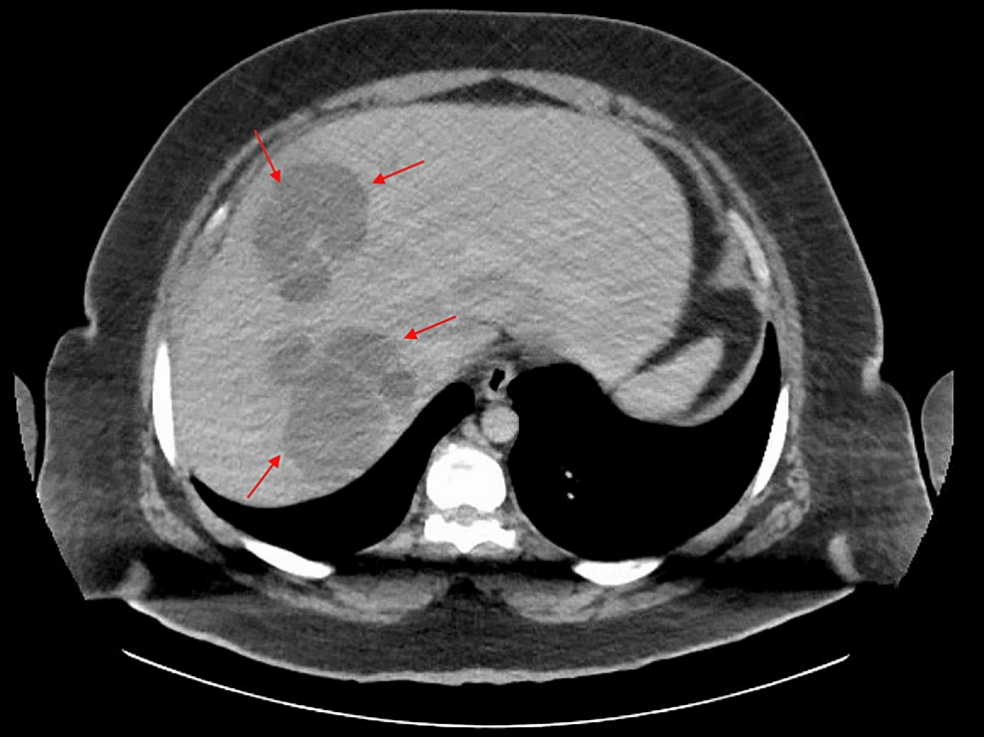
CT abdomen revealing liver lesions Red arrows point to multiloculated liver lesions

Imaging via chest X-ray showed no acute pathology, while a bedside sonogram revealed fluid pockets in the liver. The patient was started on ceftriaxone and piperacillin/tazobactam in the emergency department, which was later switched to ceftriaxone and metronidazole. CT of the abdomen with IV contrast showed hepatomegaly, measuring 24 cm in craniocaudal dimension, multiple large hypodense, multiloculated lesions throughout the right lobe of the liver, the largest located in segment 5/6, measuring 12.7 x 10.0 x 13.6 cm transverse by AP by craniocaudal with normal hepatic and portal veins and no internal gas loculations (Figure 1).

It was deemed as a likely liver abscess, with a mass being an unlikely possibility due to lack of biliary dilation and no mass effect. CT also showed mild wall thickening of the distal ileum and cecum.

Blood cultures drawn on presentation grew *Streptococcus orallis *and *Streptococcus mitis* VGS on day four of admission, which was unable to grow for susceptibility testing. The patient underwent transthoracic echocardiography to rule out infectious endocarditis, which was negative. The patient was noted to have a persistently elevated international normalized ratio (INR) which peaked at 2.44, and was subsequently started on oral vitamin K supplementation titrated from 2.5 mg daily to eventually 10 mg, which was continued daily. The patient was also found to be coronavirus disease 2019 (COVID-19) positive and was started on remdesivir. 

Ultrasound-guided hepatic drain placement was performed by interventional radiology on day three of admission, draining a significant amount of thick puss-like fluid. Three 10F pigtail catheter drains were placed on multiple abscess sites on the liver, and 20 cc of green puss-like fluid was aspirated from the drain and sent for culture and microbiology. The aspirate abscess culture was later shown to be positive for VGS *Gemella* species.

The procedure was well tolerated, and the patient had no associated complications. The patient was continued on ceftriaxone and metronidazole for two weeks and switched to oral amoxicillin/clavulanate. Prior to discharge, the patient was hemodynamically stable and afebrile with down-trending WBC with improvement in the size of lesions on repeat imaging (reduced from 12 cm to 4.6 cm). 

Repeat outpatient imaging a month later showed an additional 4.5 cm fluid collection in the hepatic dome, which has not been addressed. However, a laboratory workup revealed improving anemia with a hemoglobin of 12.9 g/dL, improving leukocytosis with a white blood cell count of 10.56, platelets 370 K/uL, normal BUN creatinine, and normal liver enzymes as well as ALT, AST, alkaline phosphatase, bilirubin, prothrombin time (PT), partial thromboplastin time (PTT), and INR.

## Discussion

PLAs are more prevalent in developing countries where the etiology directly extends to hepatobiliary or intestinal infection [5]. *E. coli *and *Klebsiella* are the most frequently isolated pathogen in PLA cases in the United States. *Streptococcus viridans*, a rare causative agent, has been identified in the case presented here.

Common presenting symptoms of PLA include chills (78%), malaise (53%), and anorexia (49%). Patients may also experience right upper quadrant pain, tenderness (35%), hepatomegaly (18%), or an enlarged liver. Septicemic shock, a life-threatening condition, can also occur in some cases (18%). Jaundice (14%) and weight loss (10%) are less common symptoms. Laboratory findings in patients with PLA may include anemia level, with 76% of patients having a low level (11.6 ± 1.9 g/L) and leukocytosis in 84% of patients having a high level (17.2 ± 6.6 x 10^9/L) hypoalbuminemia has also been found in 94% of cases [6]. Diagnosis is typically established through radiological imaging, such as ultrasonography or CT scans. The cornerstone of treatment is a combination of antibiotics and percutaneous drainage by interventional radiology, which proves effective for most patients, with operative drainage reserved for severe cases [4,6,7]. A study of 80 patients over a three-year period with PLAs over 5cm demonstrated that surgical drainage provides better clinical outcomes than percutaneous drainage for liver abscesses that are 5 cm or larger [7]. However, more recent studies have called into question the need for surgical drainage even for larger abscesses [8]. A meta-analysis of 11 studies, including 2375 patients in East Asia, found the mortality rate of *Klebsiella* PLAs to be 3.9% and non-*Klebsiella* PLAs to be 7.6% [9].

We report an unusual case of a complicated, isolated PLA in a patient without known comorbidities who was born and raised in the United States with no recent travel history. A CT scan with contrast revealed multiple hypodense multiloculated lesions in the right lobe of the liver, measuring up to 13 cm, with mild wall thickening of the distal ileum and cecum. The abscesses were later confirmed as VRS *Streptococcus viridans* PLA.

This case emphasizes the importance of considering liver abscess as a differential diagnosis even in previously healthy individuals with no known comorbidities, as early recognition is crucial for preventing morbidity and mortality. The rarity of *Streptococcus viridans* as a causative agent in PLA cases in developed countries further highlights the need for a high index of suspicion in patients presenting with nonspecific abdominal symptoms.

Treatment for PLA generally consists of antibiotics and percutaneous drainage by interventional radiology, which has been effective in the majority of patients. The patient was treated with ultrasonography-guided drainage and IV antibiotics, leading to a swift recovery and subsequent discharge. The size of the abscess, in this case, may have contributed to the decision for ultrasonography-guided drainage.

## Conclusions

This report presents a rare and unique case of a complicated, isolated PLA caused by vancomycin-resistant *Streptococcus viridans *in a previously healthy individual with no known comorbidities and no recent travel history. The patient was successfully treated with CT-guided drainage and IV antibiotics, resulting in a quick recovery and discharge. This case highlights the importance of considering liver abscess as a differential diagnosis, even in healthy individuals. It emphasizes the need for a high index of suspicion when patients present with nonspecific abdominal symptoms. Furthermore, the rarity of *Streptococcus viridans* as a causative agent in PLA cases in developed countries underlines the need for vigilance in identifying unusual pathogens. In this case, the size of the abscess may have contributed to the decision for ultrasonography-guided drainage, as larger abscesses have been shown to have better clinical outcomes with surgical drainage. Overall, this case underscores the significance of prompt recognition and appropriate treatment of PLAs in preventing morbidity and mortality, even in patients with no known comorbidities.
